# Mapping the postwar legacies of eugenics in socialist countries: a conceptual history of eugenics in Hungary

**DOI:** 10.1007/s11019-024-10218-7

**Published:** 2024-07-16

**Authors:** Péter Kakuk, Judit Sándor

**Affiliations:** Center for Ethics and Law in Biomedicine, Central European University, Nádor u. 9, FT 708. 1051, Budapest, Hungary

**Keywords:** Eugenics, Conceptual history, Postwar period, Socialism, Hungary

## Abstract

The paper aims to understand the various legacies of eugenics in the postwar period to recognize both the continuities and discontinuities of eugenics with an approach which is both conceptually sound and historically correct. Building on earlier work of Lene Koch, the paper endeavours to chart the historical trajectory of eugenics by examining how its definition and those of its related or oppositional concepts have evolved within selected lexicon entries across various stages of the century. The inclusion and publication of a concept within a lexicon indicate its significance, linguistic vitality, and prevalence in public discourse. These entries serve as a window into the contemporary understanding and application of concepts over an entire century, offering insights into the practices of eugenics as interpreted by the authoritative scholars of the era. Additionally, these lexicon entries offer more than just a mirror to the past’s prevailing attitudes. The very act of articulating a concept may be viewed as a pivotal element in social struggles, influencing the course of eugenic practices and their interpretations. Both conceptual history and discourse analysis share common ground in their perception of concepts, considering the use of language as a social activity endowed with performative capabilities. They recognize that language does not merely reflect reality but can actively shape it, playing a significant role in societal dynamics and power relations. The Hungarian lexicon entries on eugenics reveal notable disparities in the identified content, the periodization, and the evolution of changes when compared to Lene Koch’s earlier study on Scandinavian eugenics. In Hungary, the concept of eugenics underwent significant changes over four successive periods. The history and interpretation of eugenics can vary significantly from one country to another. Different nations have had their unique experiences and trajectories with the eugenics movement, which have been shaped by their specific cultural, political, and social contexts. These variations emphasize the importance of considering the localized and historical perspectives when examining the concept of eugenics.

## Introduction—postwar legacies of eugenics

Eugenics has a diverse, long history that travels through the twentieth century, across continents and countries, finding home in different institutions, accommodated by various ideologies, embedded into public health and medical practice. Eugenics was able to provide a framework for utopian ideas, but also connected to dystopic consequences. American eugenics, Nazi eugenics, British eugenics, Scandinavian eugenics, and Latin American eugenics are all rooted in Galtonian ideas but took very different forms and opened up different social practices (Kevles 1986; Adams 1990; Rose 2007). In the early years of the cold war period, it might have seemed that eugenics is irrevocably discredited and stigmatized. However, there were clear examples for both the continuities and discontinuities of eugenics in the postwar period. (Cavaliere 2018) Eugenic journals were still alive and publishing academic papers after 1945, even some new journals were being established (like the Eugenics Quarterly, in 1954). Some eugenic societies were still functioning, and some of their controversial laws were still being enacted e.g., the sterilization laws in Scandinavian countries (Dijkötter 1998; CRR, Center for Reproductive Rights 2003). The formal, organized eugenics movement lost substantial credibility and support following World War II, especially after the revelations of the Nazi atrocities, but eugenic thought did not entirely disappear. Some individuals and groups have continued to advocate for various eugenic practices well into the late 20th and even the twenty-first century, often in different forms or under different names.

In the postwar period, eugenics entered the dynamics of stigmatization (Ramsden 2009). After 1945 a discursive space started to emerge where boundary work (Gieryn 1983) intensified in demarcating genetics from eugenics, building the boundaries between good science and bad science, developing distinctions between private and public eugenics, and differentiating totalitarian from liberal eugenics. However, these distinctions to approach the concept of eugenics are often either lacking clarity or fidelity to the historical records. The implications and freedoms of state neutral eugenics are by no means clear as suggested by Diane B. Paul: “[…] *Policies are characterized as eugenic if their intent is to further a social or public purpose, such as reducing the costs or sparing future generations unnecessary suffering. Expansion of genetic services motivated by concern for the quality of the population would be eugenic by this definition, while the same practices motivated by desire to increase the choices available to individuals would not be*.” (Paul 1994, p.69) This underscores the nuanced and context-dependent nature of eugenics and related concepts. As a result of these demarcation efforts another level of diversity was introduced into the already diverse concept of eugenics and consequently introduce discussions on the different postwar legacies of eugenics.

These legacies can originate from eugenics’ connection to racial hygiene and Nazi terror, to racism and discrimination, eugenics interconnectedness with the early development of genetics or these legacies can emerge from current practices of population thinking, public health, and genetics. Fears of eugenics still play a considerable role in the public understanding of genetics. The legacy of eugenics is important for contemporary law and bioethics. Safeguards against eugenic practices raised some concerns in developing laws, guidelines, and codes of conduct in the field of biomedical science and genetics. In a way the fear of eugenics was among the catalysts of the ethical and legal norm developed around the contemporary genetics.

**Fig. 1:**
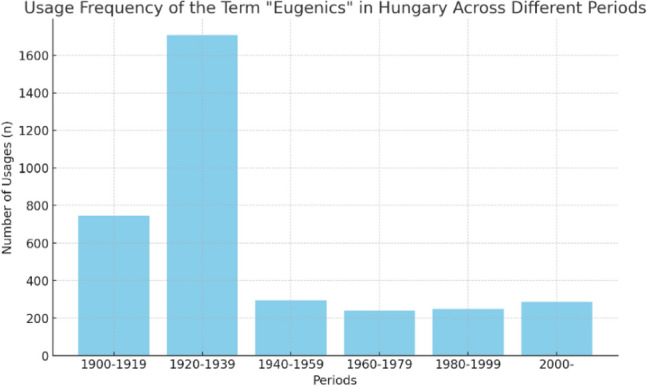
The usage frequency of the term “eugenics” in Hungary across the 20th centruy

Some legacies are easier to recognize and consider, like the dominant Nazi legacy which acts as a reminder for our present to avoid the return of history. This stigmatizing concept of eugenics has been intensively used in bioethical discourse, influencing public debates and policymaking regarding the new genetic technologies. (Cavaliere 2018) At the turn of the twentieth century through the establishment of the ELSI part of the Human Genome Project, eugenics became the „approved” project anxiety. James D. Watson, personal account on launching the Ethical Legal and Social Issues of the genome program explicitly refers to the eugenics legacy as all important aspect: *“In bringing ethics into the Genome Project so early, I was responding to my own personal fears that critics of the Genome Project would be quick to point out that I worked at the Cold Spring Harbor Laboratory, which once housed the much- controversial Eugenics Record Office Archives (ERO). If I failed to jump-start the genome ethics program, it could be used as false evidence to portray me as a secret eugenicist with the long-term goal of clearly identifying the genes responsible for social and occupational stratification and justifying racial discrimination*.” (Watson 2002, p.212) Accordingly, the understanding of these historical episodes was relevant since they demonstrated that even based on much less convincing scientific support, or in the total absence of scientific evidence, inheritable characteristics often served as a basis for the various forms of discrimination and even for eugenic sterilization, selective killing, and other often (and later) recognized crimes.(Fig 1)

Since the 1960’s new trajectories of eugenics were coming up as bioethical themes in different socially contested segments of human gene technologies, like the politics of reproduction, transhumanism, reprogenetics and fertility services, genetic testing, screening, and counselling. The emergence and widening impact of this bioethical use of the history of eugenics created a gap between normative and historical understandings of eugenics. (Anomaly 2022; Porter 2017) The normative use of the concept, in policy and bioethics, usually reduces the complexity of the history of early eugenics and its geographical and political diversity. And, at the other side of the gap, the historical understandings of eugenics are often fall short of considering the postwar continuities of eugenics. (Bashford, 2010)

Considering eugenics from the above-described historical perspective, we have more than enough reason to be cautious when using eugenics as a simple concept. As Lene Koch argues, “*the witless reference to ‘eugenics’ with no further specification is empty and more often a function of our own projections and intentions than a reference to history*.” (Koch 2004, p.329) Certainly, it demands a reflective awareness on the diversity of meanings eugenics seems to offer for a variety of audiences, especially with the often uncritical use of concepts like, “eugenic aims”, “eugenic thinking” or “eugenic values”.

This highlights the importance of writing a conceptual history of eugenics, to identify these important differences, to capture its uses, the values attached to it, its normative functions, to identify its opponent and demarcation concepts, to understand how people were thinking about and through this concept. Our aim is to understand the various legacies of eugenics in the postwar period to recognize both the continuities and discontinuities of eugenics with an approach which is both conceptually sound and historically correct. Conceptual history is not construed as a radical alternative to traditional history, but as an important and necessary supplement. Here, writing the conceptual history of eugenics is not a goal in itself but is meant as a contribution to more traditional history, to create a background understanding that provides guidance for further studies.

## Method: conceptual history as an analytic tool

In seeking to trace the evolution of the concept of eugenics in Hungary throughout the twentieth century, the paper intends to build upon Lene Koch’s investigations into the conceptual history of eugenics in Scandinavian countries by examining a curated selection of Hungarian lexicon entries from that era. (Koch 2006) Lene’s methodological insights are originated from Koselleck’s approach in the Begriffsgeschite which is concerned basically with linguistic analysis of the changing meaning of concepts. (Koselleck, 2004) More recently, others have suggested deeper and wider relevance for the place of conceptual history in constructing a critical history of the present, and as a means to theorize processes of historical change. (Müller 2014) However, this paper uses the approach of conceptual history as a simple analytic tool, where concepts are crucial for the formation and operation of society and act as a prerequisite for social and political engagement. Therefore, social and political progress can be viewed as a semantic contest to define, uphold, and ascertain the meanings of various concepts.

Histories of eugenics commonly highlight that the demographic shifts brought on by industrialization, urbanization, poverty, and scientific advancements in fields like statistics, genetics, and medicine created a novel context for interpreting the social and political environment. This context fostered the rise of eugenics as both a concept and a practice. Thus, conceptual history examines the ways in which social changes have been understood and articulated by historical actors.

The paper endeavors to chart the historical trajectory of eugenics by examining how its definition—and those of its related or oppositional concepts—have evolved within selected lexicon entries across various stages of the century. The inclusion and publication of a concept within a lexicon indicate its significance, linguistic vitality, and prevalence in public discourse. These entries are vital to understanding the common utilization of a concept and offer a concise textual corpus that facilitates an examination over an extended timeframe. Before the rise of search engines like Google, lexicons played a crucial role in language learning, writing, translation, and the study of literature.

In essence, lexicons were critical reference materials for anyone needing or wanting to ensure the correct use of language. They were particularly important when instant online lookups were not available, making them a staple in homes, libraries, schools, and offices. These texts provide an opportunity for comparative international analysis within other nations in the same region. Lexicon entries represent a highly reflexive genre, and being brief as well as easily accessible they represent what has been called a “cultivated semantics”, meaning a carefully written and thought-out definitional work on specific terms or concepts. These entries serve as a window into the contemporary understanding and application of concepts over an entire century, offering insights into the practices of eugenics as interpreted by the authoritative scholars of the era.

Additionally, these lexicon entries offer more than just a mirror to the past’s prevailing attitudes. The very act of articulating a concept may be viewed as a pivotal element in social struggles, influencing the course of eugenic practices and their interpretations. Both conceptual history and discourse analysis share common ground in their perception of concepts, considering the use of language as a social activity endowed with performative capabilities. They recognize that language does not merely reflect reality but can actively shape it, playing a significant role in societal dynamics and power relations.

### I. Progressive theorizing 1900–1920

The volumes of the first great lexicon, the Pallas Nagy Lexikona was published close to the turn of the century between 1893 and 1897, but without an entry on eugenics. Galton was regarded as a prominent figure worthy to receive an entry, but this brief section introducing Galton as an English traveler and writer, listing his major books, but without mentioning eugenics and his book on hereditary genius is neither emphasized nor discussed. The first Hungarian lexicon, the Révai Nagylexikona, which devoted a specific entry to the concept of eugenics was published in 1913. In Hungary, in the period between 1900 and 1919—as elsewhere in Europe and the USA—eugenics occupied a central place within several theories about the nation’s social and biological improvement. In 1910’s there were already two eugenic societies and a host of other institutions promoting research to improve the health of the population In Hungary. A Eugenic Committee was created in Budapest in 1914 followed by the creation of the Hungarian Society for Racial Hygiene and Population Policy in 1917.

According to Marius Turda, at these years, the country was at the forefront of eugenics in East- Central-Europe, similarly to the Czechs and Poles. German racial hygiene is often described as dominant in the region, but British, American, and French eugenics were equally influential. (Turda 2007) With the early involvement of Pál Teleki—a prominent political figure of the era, prime minister of Hungary in 1920 and in 1939—the eugenics movement political and social impact developed steadily in the country. Eugenics had achieved a mainstream political status by the end of the First World War. As Turda summarized: “*The appeal of eugenics was based upon an ambitious program, predicated upon constructing a modern Hungarian state, one characterized by a broad conception of public welfare, health care and social hygiene, as well as a nationalist vision of biological renewal. The emergence of eugenics in early twentieth- century Hungary therefore reflects this diversity of opinion, revealing striking interdisciplinary connections between various cultural and political discourses regarding the nation’s biological future then circulating*.” (Turda 2014, p.239).

The initial phase of the Hungarian eugenics movement displayed distinctive traits. In its early years, eugenics, in the vein of Francis Galton, garnered support from a spectrum of intellectuals, including progressive and leftist thinkers like József Madzsar, Lajos Dienes, and Zsigmond Fülöp. (Perecz 2005) Many from this diverse intellectual milieu were associated with the Huszadik Század journal and the Society of Social Sciences. For these individuals, eugenics was intertwined with notions of public health, social hygiene, and child welfare, yet it also existed alongside a more biological perspective of national advancement. This version of a modern Hungarian state was further advocated by health reformers such as Henrik Pach, who proposed a form of eugenics aligned with public health and social cleanliness. Therefore, the eugenics movement of the time incorporated proponents from various points on the political spectrum, including liberals, feminists, republican socialists, as well as religious conservatives and royalists. This early form of eugenics was rooted in a modern, rational, and scientific approach to social and biological betterment, managing to unite a broad array of progressive factions.

Developing the *Révai Nagylexikona*, János Mór Révay started from the great Pallas lexicon and wanted to create a modern, up-to-date lexicon containing scientific results, so he involved almost the entire Hungarian scientific world in the editing. The twenty volumes without additions contain about 1,050 sheets (17000 pages), 230000 headings, about 113 million letters and thousands of figures.

Compared to the lengthy, four columns texts which were typical in the Scandinavian entries of the same period, *Révai Nagylexikona* presents eugenics in a moderate length entry, introducing the term as a new biomedical science established by Galton. The new science is supplemented by some technologies, political measures to promote the perfection of the human race. Before describing some of these measures that could be introduced, and which might seem radical, we are reminded about an old measure that is already embedded in our laws, which is “the restriction of marriage between blood relatives.” The potential interventions on human breeding are listed as the “health examination of newlyweds, the banning of stunted people from having children, the mandatory breeding of those with excellent physical and mental qualities”. However, the conclusion is that these new measures are not realistic within the framework of “today’s social order” which is guided by a humane understanding, which holds that “*the stunted, the sick, and the mentally ill should be cared for, and criminals should be taken into state care*”. The entry imagines that these new measures might be become possible in the future, but”*only after a gradual and radical transformation*”.

American and Swiss practices on sterilization are referred to show where these measures have been already established, and some open questions are listed that must be solved by the new science. The entry closes with listing the name of the German and British pioneers of this new science, Becher, Sommer, Schalmayer, Ammon is mentioned and the major forums of the field “English and German journals of Biometrika, and the Archiv für Rassen und Gesellschaft-Biologie and the Politisch-Anthropologische Revue deal with these questions”. In this initial lexicon entry, despite acknowledging British and emphasizing German academic discourse, the topic of racial hygiene is conspicuously absent. Instead, eugenics is presented as an optimistic science with substantial potential benefits for humankind. It anticipates a possible clash with established social norms, a tension that could be resolved only with substantial societal transformation.

Thus, this entry introduces, quite early on, an explicit skepticism regarding the practicality of the agenda of negative eugenics. This skepticism is rooted in potential conflicts with humanistic values and social conventions. While eugenics had captured the attention of specific intellectual groups at the time, evidenced by thematic editions in scholarly journals that demonstrated an expanding academic curiosity in the subject, the lexicon entry addressed to the general public offered only a concise and somewhat superficial treatment of the concept.

### II. The interwar years 1920–1940

During the interwar years, the eugenic movement in Hungary underwent a significant ideological shift. Not only was it stripped of its leading advocates, but it also became confined to a more restricted and doctrinaire trajectory, influenced by the rise in nationalism and anti-Semitism that characterized the 1920s and 1930s. The conclusion of the war in 1918, followed by the successive democratic and communist revolutions, along with the counterrevolution and the ramifications of the Treaty of Trianon, signalled profound shifts that would significantly shape the evolution of eugenics in Hungary.

The Treaty of Trianon, signed on June 4, 1920, had significant and long-lasting impacts with leaving a deep and enduring mark on Hungary, shaping its national identity, politics, and relationships with neighbouring states for decades to come. Hungary lost about two-thirds of its territory as a result of the treaty. Regions with significant Hungarian populations, including Transylvania, Slovakia, parts of Croatia, and parts of Vojvodina, were ceded to neighbouring countries such as Romania, Czechoslovakia, and Yugoslavia. The treaty resulted in the forced migration of millions of ethnic Hungarians from the newly formed border areas to Hungary, leading to economic and social disruptions. Hungary lost access to valuable natural resources, industrial centres, and agricultural land due to the territorial losses. This had severe economic repercussions, contributing to economic instability and hardship in the country. Overall, the treaty had a profound impact on Hungary’s national consciousness, leading to a sense of injustice and grievance among Hungarians. The loss of territories inhabited by ethnic Hungarians created lasting bitterness and fuelled nationalist sentiments. The perceived unfairness of the treaty contributed to political instability in Hungary, fostering nationalist and revisionist movements that sought to overturn its terms. This instability had repercussions for Hungary’s domestic politics and its relationships with neighbouring countries.

The eugenic aspiration for a modern Hungarian state—one that embodied the historical entitlement and the racial vigour of the Hungarian people—was now critically compromised. However, it did not vanish entirely. Committed to its pledge of national rejuvenation and biological revitalization, eugenics resurfaced with increased vigour during the interwar years and throughout the Second World War. (Turda 2014).

The period also witnessed an increasing use of racial discourse in the country. However, the concepts of race, race hygiene, race improvement were connected to eugenics, but need to be explicated further, as these had specific meanings in this historical period of the country. (Gyurgyák, 2001, pp. 17–32.) First, race is used for Human Race (human species, Homo Sapiens) and in a plural as races which signified the different “types” or groups within the species. This latter usage and its shameful history, which gave rise to a still ongoing controversy on racist discourse generally and scientific racism more specifically. In Hungarian a different term is used for race and races, “faj” for race and “rassz” for races. Thus, the term “race” can be used without racist implications, as human race (emberi faj, Homo sapiens).

However, even in Hungarian these words are used rather ambiguously. For example, one of our selected lexicon entries devoted to the topic of the “racial question” (faji kérdés) provides a lengthy discussion of the term’s usage in the era with the following wording: “*a race is a certain group of people with physical and mental characteristics that can be distinguished from other groups. Anglo-Saxon, Latin (Romanian), Aryan (or rather Indo-Germanic), Semitic, *etc*., are used in this sense. Race, in relation to humans, is a concept that can hardly be defined scientifically, anthropology talks about types (races). However, there is no doubt that a group of people living in the same place for a long time, under the same living conditions, takes on more or less the same mental qualities, which distinguish them from other groups. There is little doubt about the correctness of the division of humanity into three large groups; and these are: the white, the yellow (excluding the red-skinned) and the black. It is all the more difficult to make a racially sharp distinction between the individual groups of people: the individual “races” are mixtures, one merges with the other*.” (Uj idők Lexikona 1938, “Faji kérdés”).

In German, the term “Rassen” can be used for both meanings. In Germany, when the term “Rassenhygiene” was coined by the early supporters of the idea to use advances in biology to improve the gene pool, it had the same meaning as eugenics. Thus “Rassenhygiene” did not necessarily referred to race based eugenics originally. It was a bit later, when the nazis took over the racial hygiene society, which “marked a shift from an inclusive biological approach to welfare to one based on race, coercion, and violence against those deemed undesirable for biological and racial reasons”. (Weindling 2010, p.321).

In the nazi racial hygiene theory during the 1930s and 1940s in Germany, the difference between the human races were scientifically established, essential and hierarchically ordered. It aimed to promote the idea of racial purity and superiority of the so-called “Aryan race” while advocating for the elimination or control of other perceived “inferior” races, particularly Jews, Roma (Gypsies), Slavs, and people with disabilities. The ideology of the Hungarian race protection movement was different, even if it was sharing some of these nazi ideological elements. Importantly, the ethnic diversity and mixture of the Hungarian people were too obvious that challenged the proofs of Aryan ancestry. According the Gyurgyák, the Hungarian race protection movement represented a special case in racial theorizing, basically diverged from the German “hierarchical listing” even if they were deeply antisemitic. The Hungarian usage of race was rather ambiguous and contradictory, far from being defined by biology, and more relying on a mythic identity of an ethnic group, sometimes using the origin myths of Turanism. (Gyurgyák, 2001, p.30.)

In the interwar years, eugenic ideologies concerning the Hungarian race found favour with political entities, including parties like the conservative Christian Social and Economic Party (), which incorporated eugenic principles into its agenda as early as 1919. The early 1920s saw the emergence of new scientific and religious bodies, such as the Hungarian Scientific Association for Race Protection [*Magyar Tudományos Fajvédő Egyesület*] and the Mission for the Saving of the Hungarian Race [*Magyar Fajmentés Misszió*], which embraced and promoted eugenic concepts. Together with professional organizations like the National Association of Hungarian Physicians [*Magyar Orvosok Nemzeti Egyesülete*] and the St. Luke Society of Catholic Physicians [*Katolikus Orvosok Szent Lukács Egyesülete*], these groups played a pivotal role in propagating an increasing eugenic rhetoric focused on the social and biological enhancement through safeguarding the family, the nation, and the race.

A draft for the eugenic sterilization law was developed and proposed by a professor of psychiatry, László Benedek who was the Hungarian Representative of the Committee of International Eugenical Organisations, but finally the sterilization law was not enacted. Benedek initiative was supported by some psychiatrists: Márk Goldberger, Lajos Naményi, Gyula Donáth. According to the opponents of eliminative eugenics the measure of sterilization had not been scientifically established, because the ways and the probability of heredity of nervous and mental diseases, except for Huntington chorea, had not been explored yet. The outstanding neurologist, Károly Schaffer also opposed the sterilization law drafted by Benedek, so it was rejected by the National Council of Public Health [*Nemzeti Egészségügyi Tanács*] in 1932 and was not discussed by the Hungarian Parliament either. (Síró, 2003) Sterilization was regarded as a drastic eugenic step, the efficacy of which, in preventing social and biological decline, was not conclusively established by science and was at odds with the moral teachings of Catholicism. Despite several attempts, the implementation of sterilization laws did not materialize. However, other forms of negative eugenics, such as mandatory medical examinations prior to marriage, were implemented in 1941. These premarital health checks were advocated as vital measures for safeguarding the integrity of the Hungarian family.

In the 1930s, Hungary saw the formation of multiple organizations like the Society for Biopolitics and the Hungarian Union for Family Protection, both of which campaigned for the integration of eugenic policies into societal governance. The establishment of the Marriage Counselling Institute [*Házassági Tanácsadó Intézet*] in 1938 was another step towards institutionalizing eugenic ideas. Prominent figures such as the anthropologist János Gáspár took the lead in these initiatives, becoming the head of the Department of Heredity, Racial Biology, and Eugenics in 1939. Subsequently, the Hungarian Institute of National Biology was founded in Budapest in 1940. That same year, the esteemed Hungarian anthropologist Lajos Bartucz was at the helm of the newly inaugurated Institute for Anthropology and Racial Biology at Horthy Miklós University in Szeged, further cementing the academic and institutional presence of eugenic thought in the country.

The editing of the *Gutenberg Great Lexikon* started in 1931 as a major undertaking of Hungarian cultural life between the two world wars. Nine large volumes with 640–670 double-columned pages were already published in 1931, followed by a tenth in 1932. At that time, however, the publication of the work still under the letter “F” was interrupted, and it was not completed later. Despite this, the volume of the material published until then reaches almost 3,300 printed pages, making it one of the largest books of the era. Thus, the Gutenberg Great Lexicon’s entry on eugenics and the related terms were published in 1932. The lexicon also devoted a section on “Racial biology” (Fajbiológia), “Racial purity” (Fajtisztaság), és “Racial protection” (Fajvédelem).

Here, “racial biology” is defined as the branch or trend in anthropology that “*studies the inherited and acquired characteristics of human races from the point of view of how racial traits are related to various environmental influences and what role they play in individual, family, social, and state life. Based on the lessons learned from such investigations, it strives to direct the development of peoples, nations, and societies in a certain direction through practical intervention*.” Interestingly, this definition of “racial biology” emphasizes acquired characteristics and environmental influences. Thus, “racial biology” will be hardly differentiated from those conceptions of eugenics, which are avoiding the reductionism of hereditarian and genetic determinist thinking.

In the entry on “racial purity”, we are informed that in the case of humans, even in the case of primitive peoples, racial purity is non-existent, and: “*race mixing in all directions is not beneficial, but it has benefited humanity more than it has harmed*.” The entry questions the basic presumptions of the nazi version of racial hygiene to provide space to an ambiguous “Hungarian version” of the race concept, which might be able to accommodate “non-biological racial ideas”, amid ethnically diverse historical realities.

Reading the entry on “racial protection” we come to learn that it is “*an effort to maintain and strengthen the beneficial physical and mental qualities of the individual, on the one hand, and on the other hand to eliminate or at least neutralize the unwanted elements. Its tools are partly the same as the tasks that promote racial hygiene, but in addition, its special concern is to awaken, keep awake and develop the racial self-consciousness of a people.”* In the lexicon, “racial protection” has this added task of enabling the ideological improvement of the people, to develop their racial self-consciousness.

In the *Gutenberg Great Lexikon*, “racial hygiene” appears to be employed without controversy and is used interchangeably with “eugenics.” It distances current eugenics from Galton with references to the eugenicists of its era who “consider negative selection more important”. State intervention in forms of compulsory marriage counselling and prohibitions of marriages and sterilization is mentioned as core elements of eugenics, but the realization of these measures is projected to the future, while “in most culture states they have so far only fought in the form of social associations in order to have their doctrines recognized”. Eugenics is acknowledged predominantly as a theory in progress, with its associated practices yet to be established and realized in the future.

The *Uj Idők Lexikon* was one of the important cultural publications of the period between the two world wars. The entire series, which was published in just 6 years, contained 24 volumes, each with 255 pages—typical for lexicons in two columns. The total volume of the truly enormous work is about 6200 printed pages and as the editors say: “*It contains around 100000 headings. We compiled these 100000 headings in such a way that they include everything that is important and interesting for the educated audience.”*

The lexicon’s entry defines “racial biology” as based in natural science, “as a branch of science that deals with the different life manifestations of the “human race” (Homo sapiens), which is thought to be uniform, according to its smaller taxonomic units, subspecies, species and even smaller units.” Then, entry follows the same approach as the Gutenberg Lexicon form the same period, but also argues that “racial biology” “*moves further and further away from the natural science base and slips into the field of the spiritual sciences and, with an even greater deviation, into the field of politics.*”

“Racial hygiene” is defined as forming a “*part of the science of health, which is again divided into several parts, such as heredity, people dealing with racial history and racial description and eugenics*”. We are informed that racial hygiene institutes are dealing with heredity research, family research, statistical group observation, and twin research, etc. and “*their results strongly influence the legislation of some states*”.

The *Új Idők Lexikona* recognizes eugenics within the broader framework of racial hygiene, perceiving it as one component of a larger constellation that includes the science of heredity, the history of race, and racial description. In this view, eugenics is situated as a subfield or a complementary aspect of racial hygiene rather than a standalone concept or practice. The entry refers to American and German eugenic laws, and law against miscegenation.

It’s notable that the lexicon entry, when enumerating potential initiatives under the banner of positive eugenics, doesn’t solely focus on biological or medical interventions. Instead, it includes a variety of social taxes and policies. This suggests a broader and more integrated approach to eugenics, one that incorporates socio- economic strategies alongside more traditional eugenic methods. Such an approach implies that the promotion of desired traits or the improvement of the population’s health could also be addressed through social reforms and fiscal measures, not just through direct genetic management. The entry suggests a eugenic “*reform of the tax system on a family basis, regulation of inheritance in such a way that only several children can inherit the property of the parents, or tax-free family and marriage loans*”. The conceptual framing in this instance paradoxically positions eugenics as both a specific subset of racial hygiene and also as a broad field that extends into policy interventions potentially influencing personal reproductive choices. This dual characterization suggests that while eugenics is seen as part of the larger enterprise of racial hygiene focused on the health and composition of a population, it is also understood to encompass a wide array of public policies that could indirectly shape demographic trends through their impact on individual decisions regarding childbearing.

The six volumes of *Uj Lexikon*, were published in 1936 and introduced as the universal encyclopaedia of knowledge and practical life. As the preface describes it: *“…with the help of excellent experts and scientists, we compiled a completely new heading material for the UJ LEXIKON. Our goal was to provide a comprehensive, objective picture of living knowledge—whether it concerns the present or the past—covering all problems*.” *Uj Lexikon* maintains a concise treatment of eugenics, employing it synonymously with racial hygiene. This suggests a continued conceptual overlap where eugenics is not distinctly separated from the broader notion of racial hygiene but rather is presented as an equivalent term within the lexicon’s entries. According to the entry, Galtonian eugenics was promoting positive eugenics, while today the “principle of negative eugenics” is more strongly emphasized, which is to prevent the creation of offspring of reduced value.

In this case, the lexicon redirects the reader from a brief entry on eugenics to a much more extensive and complex discussion under the topic of racial biology. This suggests that the lexicon may treat eugenics as a component or aspect of the wider and perhaps more scientifically grounded field of racial biology, which typically examines the biological variations among human races. The entry starts with saying that in the era following the First World War,”*the disease ravaged to a greater extent, the resistance of exhausted people decreased, and racial mixing began, which threatened the purity of the races more and more*”. Referring to Gobineau, Nell, Giddon and Ehle, the dangers of degeneration are explained, “*which may have internal causes, but which, according to species biologists, can mainly be traced back to the unreasonable mixing of species*.” The industrialization of a nation makes it difficult “*to create a healthy population and adequate viable human resources, because it provides a favourable environment for alcoholism, tuberculosis, the spread of venereal diseases and moral degradation.*” The importance of environmental influences and adaptation is also recognized, but the outmost importance of blood is emphasized.

Nearly half of the text is devoted to a discussion on blood groups and racial mixing that starts with the observation:”*The basis of everything is the composition of the blood. How different the blood of different individuals is proven by the example of blood transfusion, from which it is clear that the blood serum of an alien individual dissolves the foreign red blood cells and has a toxic effect on the body with its protein, causing a high fever*.” The presented conclusion suggests a perspective that crossbreeding among species, or race mixing in the context of human populations, is viewed negatively in terms of eugenics and breeding practices. It emphasizes that individuals have obligations that extend beyond the personal scope to encompass duties toward their race. In this view, disregarding these responsibilities is framed not merely as a personal failure but as a grave societal offense. Such a standpoint intensifies the weight of individual reproductive choices, casting them as matters of social and racial consequence and labelling race mixing as an act detrimental to society.

While the entry does articulate a stance that discourages race mixing from a eugenic perspective, it also conveys a sense of ambivalence about the practicality of implementing a racial biology program. It acknowledges the complexity of the human condition, recognizing that such extensive intermixing has occurred that the existence of “pure races” is, at best, doubtful. This reflection suggests an awareness of the intricate reality of human genetic diversity, implying that any initiatives in racial biology must consider the current state of human genetic intermixture. The entry’s cautious tone indicates a recognition of the challenges inherent in applying theoretical eugenic or racial purity principles to the nuanced and blended tapestry of human populations.

Moreover, according to this entry on racial biology, “*unrestricted mixing of blood is the ruin of the race, but let’s not forget that even among individuals belonging to mixed races there are often very excellent ones*”. The entry goes along an unusually lengthy discussion of racial science with a strong emphasis on blood groups and racial mixing but involves only some scant commentaries on marriage laws and policies that are or could be based on the science. Premarital health examinations were seen as essential for the protection of the Hungarian family, but still, state intervention into traditional marriages regarded as highly contested regarding its practical feasibility. The entry’s general approach is rather cautious especially in drawing its final conclusions and becomes explicitly sceptical regarding the feasibility of racial biology’s mission in intervening into the accepted procedures of marriages. “*Regulating this by law would be the most sensitive intervention in human life.*” The circumspect position in the lexicon entry is particularly striking given its temporal proximity to the enactment of marriage laws that forbade unions between Jews and non-Jews in Hungary, the so-called law (Act XV of 1941) prohibiting marriages between “Gentiles and Jews”. (Szikra 2008; Kovács 1994).

### III. Postwar period 1945–1990

The second world war and the interwar years both had a devastating effect on the country’s institutional infrastructure and resources. In the early 1950s Hungary underwent a troubled transition from an independent country to Soviet occupation and ultimately to the transformation into a communist satellite state. Most of the experts and supporters of eugenics have already died before the 1950’s, or emigrated from the country earlier, some of them became marginalized or stayed away from public positions. (Turda 2015, 212) In the postwar period, the potential continuities and discontinuities of eugenic thought could be present in disciplines like human genetics, anthropology, psychiatry, psychology and education. These were the fields where ideas originated from eugenic thinking could be circulated. Those Hungarian eugenicists who were not portrayed as scientific racist continued to promote their ideas after the war, especially medical doctors, and particularly with respect to marriage counselling, which continued, but remained unpopular. (Szegedi 2012) Examples for a continuity of professional careers is Naményi who managed to reformulate his ideas in the field of marriage counselling and Malán, who continued to publish in field of anthropology until the 1960’s. (Turda 2015, p.212).

Alison Bashford’s observation of a general reluctance among historians to explore the topic of eugenics post-1945 reflects a broader trend in the historiography of the subject, where the focus tends to be on the pre-World War II era. (Bashford, 2010) Hungary fits within this pattern, with limited scholarly attention given to the continuance of eugenic thought in the postwar period. Among the sparse research in this area is Barna Szamosi’s study, which stands out for its examination of eugenics in Hungarian medical dialogues during the early years and how these ideas transitioned into the new age of genetics in the 1960s and 1970s. What makes Szamosi’s work particularly significant is the methodology—conducting interviews with contemporary geneticists to discern how eugenic language and thinking persisted in the medical discourse of the time. This approach underscores the subtleties of how eugenic ideologies were, and perhaps still are, woven into the fabric of scientific conversation, even as the explicit use of the term ‘eugenics’ has fallen out of favor. By directly engaging with modern practitioners, Szamosi’s research provides a unique perspective on the lingering traces of eugenic thought in the practice of medicine and genetics, long after the height of the eugenics movement. (Szamosi 2018, 2013).

Gábor Szegedi’s research offers a fascinating lens on marriage counseling in Hungary, examining it through the theories of biopolitics. This study uncovers how marriage counseling as a policy and practice continued into the 1950s, maintaining consistency not just in the personnel who administered it but also in its objectives and underlying principles. What stands out in Szegedi’s analysis is the remarkable durability of this aspect of public health policy, which was closely tied to the pronatalist and eugenicist ideals of the Horthy regime from 1920 to 1944. The fact that these policies and practices were able to persist into the postwar period, seemingly without fundamental alteration, suggests that certain elements of the eugenic agenda were adaptable to the shifting ideological landscapes. It indicates that despite the profound political and social changes that occurred after World War II, including the establishment of communist rule, the infrastructure and ethos of marriage counseling could be co-opted and repurposed to fit the new government’s public health objectives. (Szegedi 2012).

Marriage counselling continued with the same intellectuals and doctors without much interruption in 1945. In contrast, the area of sexual counseling, which had previously been championed by left-wing parties, liberals, and medical experts during the 1930s, did not experience a similar revival in the postwar era. The marriage and sex counseling centers that were voluntarily established in Austria and Germany during the 1920s served as the prototypes that Hungarian experts sought to emulate.

The voluntary marriage counseling centers established in the 1920s and 1930s were predominantly managed by a select group of physicians who specialized in the fight against venereal diseases (VD). This elite group of doctors formed a tight-knit community focused on addressing and curtailing the spread of VD through these centers. While the primary focus of many of these centers was on venereal disease, some extended their services to encompass a broader range of marital health issues. A number of these centers also had explicit eugenic objectives, promoting what they saw as healthier and genetically superior offspring through the advice and information they provided to couples. This indicates that marriage counseling, at that time, could serve multiple purposes: it was a means to combat public health issues like VD, as well as a platform to encourage eugenic principles under the guise of promoting marital health and family planning. The counselling either meant medical examinations (on the spot or forwarding the “patient” to a specialist) or providing public health- related advice on marrying options and illnesses, with particular attention to those that could affect one’s future children.

The law that eventually introduced marriage counselling in 1941 (Law No. XV. of 1941) focused only on the two mostly feared contagious diseases: VD and tuberculosis. The law was in effect from 1942 to 1952 except that the anti-miscegenation clause was cancelled as early as March 1945. According to the data at hand, approximately 2–4% of the 100000 to 150000 individuals who sought to marry each year were subjected to a temporary denial of their marriage applications. These obligatory marriage counselling sessions were not only about indicating whether the individual had TB or any form of VD, but the doctors also tried to use it to build up a eugenic register for future use. (Szegedi 2012).

The first significant lexicon series that was published in the period after the Second World War is the Új Magyar Lexikon from 1960. The preparatory works around these volumes started much earlier, but the 1956 revolution brought some unexpected challenges to finalize the publication of the volumes according to the original plan. After November 1956 an investigation was launched against many external members of the editorial board, and others became ideologically uncertain. It became obvious that it will be not possible to talk about certain phenomena, persons, etc., after the revolution, to write again and the same as before, even some works had to be started from scratch. Thus, the editorial work of the Új Magyar Lexicon was suspended, with reference to “mainly economic difficulties”. As György Bernát and Máté Kovács, who also edited a brief version of the series, written that the first ideological and ideological confusion of 1956, the lexicon must correctly reflect “the worldview of our people and our state, the current aspirations of Hungarian society moving towards socialism”. (Pogány 2012).

In 1945, the Hungarian eugenics movement experienced a significant disruption on multiple fronts, including human, institutional, financial, and political resources, which amplified the discontinuity of eugenics within the country. During the 1950’s, when international eugenics was entering into the “dynamics of stigmatization”, the once robust social movement already lost its strength and social resources. In Hungary, after the 1950’s, any attempts on continuation of the eugenics movement would be deterred by the totalitarian state with its Marxist-Leninist’ ideology. As the country underwent a transformation into a communist satellite state, the “bourgeois” science of eugenics, especially its Malthusian roots, and more generally its biologism was deemed incompatible with the new scientific ideologies imported from the Soviet Union.

Unsurprisingly, the eugenics entry of *Új Magyar Lexicon* introduces a significant change in language in describing eugenics as “a pseudoscience widespread in capitalist countries”. This is notably a dual demarcation process, as it not only definitively associates eugenics with non-scientific principles but also places it within the context of other countries, particularly those with capitalist systems. This act of geographical demarcation effectively separates Hungary from eugenics, creating the perception that Hungarian science is unrelated to eugenics. Instead, it links eugenics with race theories, imperialism, and references events such as Nazi concentration camps and restrictive marriage laws in the USA and South Africa. The main goal of eugenics is described as “*to cover up the fact that social conditions have a decisive influence on the development of a person’s character and mental abilities, but no less on improving their health*”.

The *Orvosi Lexikon* of 1969 defines eugenics as an application of genetics, which follows Galton’s ideas for “improving the human populations (races)”. The Orvosi Lexikon introduces the distinction between negative and positive eugenics, where the latter is described as aimed to “*promote the reproduction of individuals valuable to human society and to preserve their beneficial hereditary characteristics*”, whereas the former “*recommends the elimination of an individual from reproduction, who regarded as harmful to society [e.g., suffering from hereditary diseases]*.”

As a significant component in formulating the concept, the *Orvosi Lexikon* distinguishes between good and bad eugenics, thereby striving to establish a clear demarcation by defining a negative variant of eugenics. Bad eugenics is characterized as an “unscientific and reactionary trend” within eugenics that asserts the biological and spiritual inequality among humans and advocates for the compulsory sterilization of individuals deemed to be “less valuable.” Bad eugenics played a major role “in Germany during Hitler’s fascism” and “today” it is advocated by some “ideologues of imperialism”. Indeed, the entry raises curiosity about how “good eugenics” might be defined, as it focuses primarily on delineating the negative aspects of eugenics.

The reference to “eugenic indication” and the suggestion to consult the entry on “abortus arteficialis” indicate a specific context of usage. It implies that “eugenic indication” may relate to the practice of selective abortion or artificial abortion in the context of eugenics. To gain a more complete understanding of this term and its application, one might need to refer to the entry on “abortus arteficialis” for further information and context. As that entry discusses the indications of induced abortion, the list defines the use of “eugenic indication” with reference to the fetus to be born with severe impairment. The postwar utilization of eugenics within medical terminology represents a possible legacy of eugenics. This legacy is cautiously demarcated from the unscientific and “reactionary” aspect of eugenics mentioned earlier in the entry.

### IV. Post-transition years, 1990 –

The fall of communism created a significantly favorable political climate for the free publication of books. Consequently, renowned encyclopedias such as Britannica and Larousse were published, and Hungarian versions were developed from them. The *Magyar Nagylexikon* marked the first original lexicon published after the transition. In its entry on eugenics, it provides a concise definition, describing it as the application of genetics “to enhance the hereditary traits of human populations.” Additionally, it attributes the term’s origin to Sir Francis Galton, recognized as its founding figure.

Without being very informative the distinction between positive and negative eugenics is mentioned. The pseudo-scientific version of eugenics is connected to Nazi terror, with the claim that “*unscientific use in Germany between 1933–1945 played a role in the death of millions of people—declared worthless*”. Compared to the language used by the other lexicons published in the 1960’s there are scant differences, except that imperialist and reactionary forces are not mentioned. Another great lexicon the *Révai Új Lexikona* that has been published after the transition does not contain any entry on eugenics.

The other lexicons during the transition period were predominantly translations or slightly altered versions of established English, German, and French encyclopedias, sometimes incorporating original content as well. The 1996 *Hungarica Britannica* provided a more extensive examination of eugenics, defining it as a scientific discipline that utilizes genetics to enhance the human species. The entry also adopts a historical approach to introduce eugenics to its readers, providing a broader perspective on the subject. Situating eugenics in a historical perspective, where eugenics find some roots in Plato’s work, the idea matures in Galton’ writings, and became institutionalized with the establishment of the American Eugenics Society in 1926. These developments opened up a series of policy implications that existed till the 1970’s. Criticism appeared at the 1930’ that lead to the general rejection of eugenics, but with further developments in prenatal diagnostics “the goals of eugenics—the identification and suppression of unwanted genetic material—regained their validity”. Contrary to the lengthy and subtle historical discussion of the Hungarica Brittanica, both the Magyar Larousse (1994), and the *Officina Egyetemes Lexikon* (1994) had a very simple two-sentence entry which clearly identified eugenics with Nazism.

Eugenics seems to have been a subject of exceptional focus for a single author, Dr. Endre Czeizel, starting in the 1970s. This intense interest in eugenics continued into the transition period, with Dr. Czeizel publishing entire volumes dedicated to the topic. As a prominent figure in the fields of medical genetics and medical history in Hungary, Czeizel produced works during this period that displayed an explicit eugenic orientation, like his study on the “Genealogical evaluation of the greatest Hungarian poets”, which follows Galton steps and attempts to demonstrate that talent have strong genetic components and express the hope that “*the human genome programme will provide data for the explanation of different types of genius and eminence*.” Interestingly, despite Dr. Czeizel’s writings and his explicit eugenic approach, it appears that his usage of eugenic terminology and concepts did not trigger normative discussions in Hungarian public discourse regarding the appropriateness of interpreting the legacy of eugenics in such a manner. As Turda observes, the “Czeizel phenomenon” can be seen as a clear indication that there is a lack of cultural memory of eugenics, both as a practice and an ideology, within Hungarian historiography. (Turda, 2015) Moreover, Czeizel’s use of eugenics and generally the normative questions regarding science remained mostly undiscussed by humanities scholars and social scientists as a result of the strong separation of natural science and social sciences in the cold war and also in the post-transition period in the country.

## Conclusions

The Hungarian lexicon entries on eugenics reveal notable disparities in the identified content, the periodization, and the evolution of changes when compared to Lene Koch’s earlier study on Scandinavian eugenics. In Hungary, the concept of eugenics underwent significant changes over four successive periods. The first period, which occurred in the 1910s, can be characterized as a time of progressive theorizing, marked by considerable optimism about eugenics as a means of social renewal. In the second period, spanning from the 1920s to the 1940s, which corresponds to the interwar years, the importance of the concept grew significantly, and it began to be employed as a synonym for racial hygiene. Nevertheless, both the first and second periods shared a sense of skepticism concerning the practical implementation of state intervention in reproductive choices, particularly when it came to negative eugenics. The postwar period from 1945 to 1990 brought about a radical transformation in the concept of eugenics. These evolving concepts, as derived from lexicon entries spanning different periods, serve as a valuable source for understanding the general usage and evolution of this concept.

When examining language usage, it’s important not only to consider variations in meanings across different periods but also to consider differences in the frequency or intensity of usage during these periods. This can provide valuable insights into how the concept evolved and its prominence in various contexts over time. Indeed, analyzing historical datasets of periodicals, journals, and newspapers can be a valuable approach to studying the changing intensity of language usage and the evolution of concepts like eugenics over time. Such data can provide quantitative insights into how often the term was used, in what contexts, and how its usage patterns shifted in different periods, complementing the qualitative analysis of lexicon entries and other written sources. According to data from the Arcanum platform, the term eugenics had two intensive usage periods in Hungary (1900–1919 = n.744, and in 1920–1939 = n.1708) and the frequency of the term’s usage decreased afterwards (1940–1959 = n. = 293; 1960–1979 = n.240; 1980–1999 = n.248; 2000- = n.286). (www.arcanum.hu) (Fig 1).

The early period of eugenics in Hungary could be characterized as an endeavor to theorize about how to enhance the quality of the population by applying the new insights of biomedical science. During this early stage, racial hygiene may not have been considered problematic, but there was still hesitation regarding the potential measures that might be necessary to achieve the objectives of eugenics. They perceived the practical applications of eugenics to be overly radical. It seemed that the prevailing social order was not prepared to embrace state interventions into conventional marriages. A notable characteristic of the Hungarian concept of eugenics, which persisted in both the early and interwar periods as evident in all lexicon entries, is the issue of feasibility associated with eugenics. This feasibility problem was emphasized even in the most enthusiastic and optimistic entries on the subject. In contrast to Koch’s analysis of Scandinavian lexicons, which often featured in-depth discussions spanning four columns and introduced subtle distinctions or multiple perspectives on the topic, all the prewar period entries in the Hungarian lexicons were of moderate length. This suggests that eugenics was a subject that was considered necessary to cover in Hungarian lexicons, but its level of interest and popularity had limitations, even during the prewar period.

Before the outbreak of the Second World War, the entries in Hungarian lexicons seldom made a clear distinction between racial hygiene and eugenics. Instead, they frequently used these terms as direct and unambiguous synonyms, referring to both a German and an English term for the same concept or phenomenon. In those rare instances when distinctions were discussed between eugenics and racial hygiene in Hungarian lexicons, racial hygiene was typically portrayed as the broader concept that encompassed eugenics as one of its components or aspects.

The discontinuity of the eugenics movement after the postwar period was primarily driven by the political and ideological changes in the country, which led to the dissolution of institutions and organizations associated with eugenics. Additionally, many influential advocates and experts of the eugenics movement either passed away during the first two periods or emigrated from the country.

At this point, the evolution of the concept of eugenics underwent a transformation, evolving into covering a predominantly negative, pseudoscientific, imperialist, and racist ideology. A notable exception to this transformation is found in the *Orvosi Lexikon* (Medical Lexicon) from 1969, which seeks to differentiate between positive and negative aspects of eugenics. This suggests a continuation of a particular legacy of eugenics within medical language, even amid the overall shift towards a more negative view of the concept.

The post-transition period after 1990 does not show much interest in eugenics, as lexicon entries of the era well demonstrate. It appears that eugenics is not considered an especially engaging or socially significant phenomenon for Hungarians. There seems to be limited historical research or normative discourse surrounding the topic in Hungary. Comparatively, in the United States the mainstream eugenics movement in the early twentieth century sought to control the reproduction of people of color, the so-called”feeble minded” (such as in the case of Buck v. Bell), and recently immigrated populations through sterilization and the legalization of birth control. The eugenic movement took a different form in Hungary. Hungary did not have colonies and immigrants from overseas (until 2015), and its population was a typical Central European mixture over the centuries. And after the first world war it became even less heterogeneous from an ethnic perspective. Since then, the target of eugenic practices has been certain ethnic minorities (predominantly Roma women) and people with mental disorders. Some of these eugenic sentiments are still present in Hungary, but mostly take the form of indirect nudges. There is a hidden agenda, for example, behind the social policy that supports middle and upper middle-class families by providing a generous financial aid to them in form of significant tax reductions or housing assistance in case they have three children. People from the Roma population, on the other hand, are rarely able to make use of these opportunities, as the precondition of applying for these financial aids is steady high income.

Our analyses demonstrate how lexicon entries can serve as valuable tools for comprehending the multifaceted meanings inherent in the concept of eugenics. These entries can provide insights into how these meanings evolved over the course of a century and how these changes were linked to broader social and political developments. The history and interpretation of eugenics can vary significantly from one country to another. Different nations have had their unique experiences and trajectories with the eugenics movement, which have been shaped by their specific cultural, political, and social contexts. These variations highlight the importance of considering localized and historical perspectives when examining the concept of eugenics and its diverse legacies.
